# Supplementary material for: Cryo‐EM structure of metazoan TRAPPIII, the multi‐subunit complex that activates the GTPase Rab1
Source: EMBO J. 2021 May 21;40(12):e107608. doi: 10.15252/embj.2020107608 (PMC8204870; doi:10.15252/embj.2020107608)
Supplement: Supplementary file 1 — Appendix [file EMBJ-40-e107608-s004.pdf]

Appendix

Table of Contents:

Appendix References
Appendix Figure S1. Negative staining and initial models.
Appendix Figure S2. Angular distribution of non-tilted TRAPPIII and miniTRAPPIII.
Appendix Figure S3. Cryo-EM structure of TRAPPIII and miniTRAPPIII.
Appendix Figure S4. TRAPP subcomplexes.
Appendix Figure S5. Cross-links and the TRAPPIII structural model.
Appendix Figure S6. Movement of the TRAPPC8 and TRAPPC11 arms relative to the core.

Appendix References

Nakane T, Kimanius D, Lindahl E & Scheres SH (2018) Characterisation of molecular motions in cryo-EM single-particle data by multi-body refinement in RELION. *eLife* **7:** 1485

Naydenova K & Russo CJ (2017) Measuring the effects of particle orientation to improve the efficiency of electron cryomicroscopy. *Nat Commun* **8:** 629

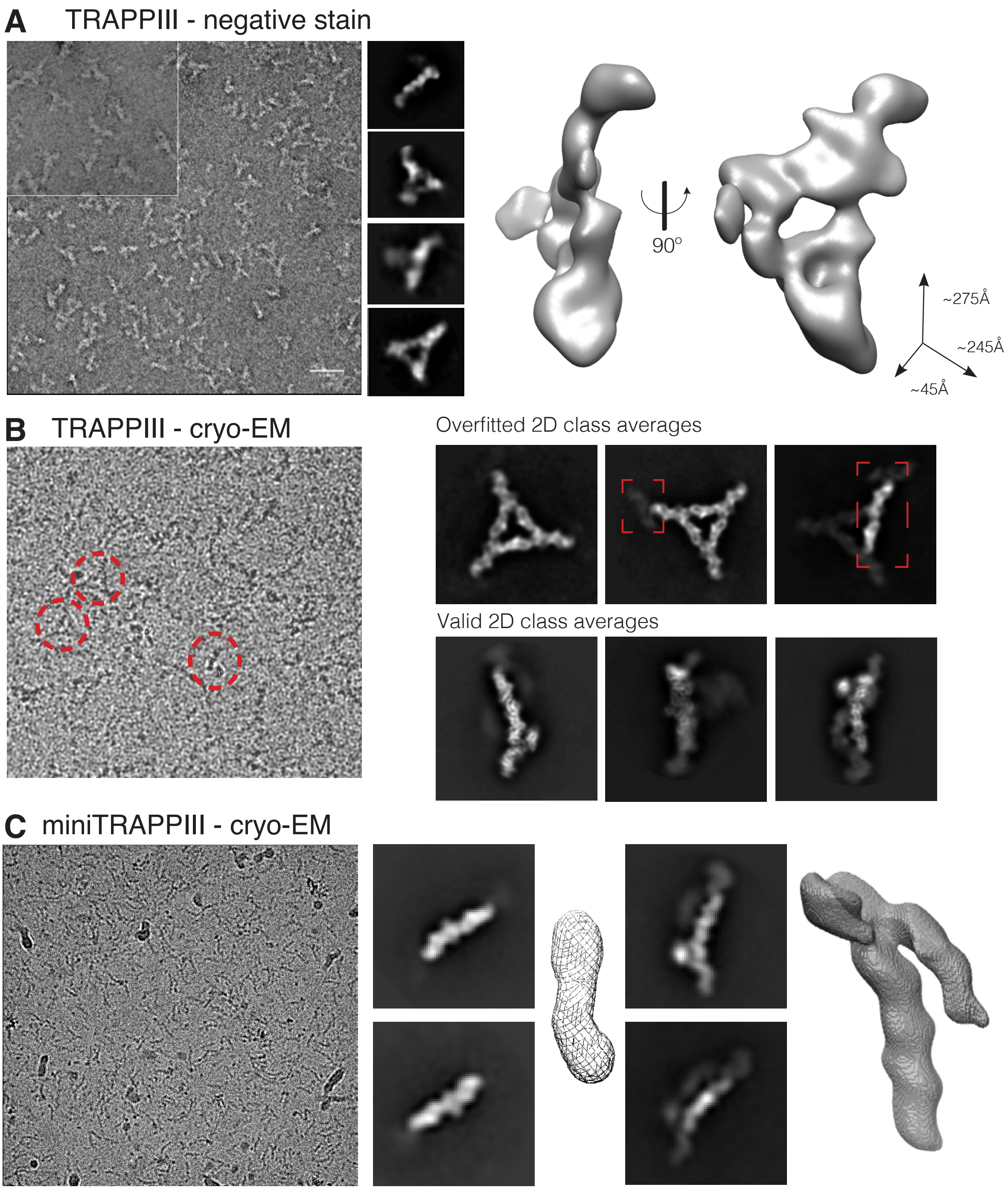

Appendix Figure S1. Negative staining and initial models.

(A) Left: EM negative staining micrograph showing TRAPPIII particles. A zoomed inset with different contrast is shown in the top left corner of the micrograph. Scale bar 50 nm. Middle: Representative 2D classes. Right: 3D model from negative staining 2D classes.

(B) Example cryo-EM micrograph of the whole TRAPPIII complex. The different particles shapes are highlighted with dashed circles. Overfitted and real 2D class averages are shown, with two of the latter (left and right) also shown in Fig 1C, and included here to aid comparison with the overfitted averages.

(C) Example cryo-EM micrograph of miniTRAPPIII. 2D class averages and 3D initial models are shown.

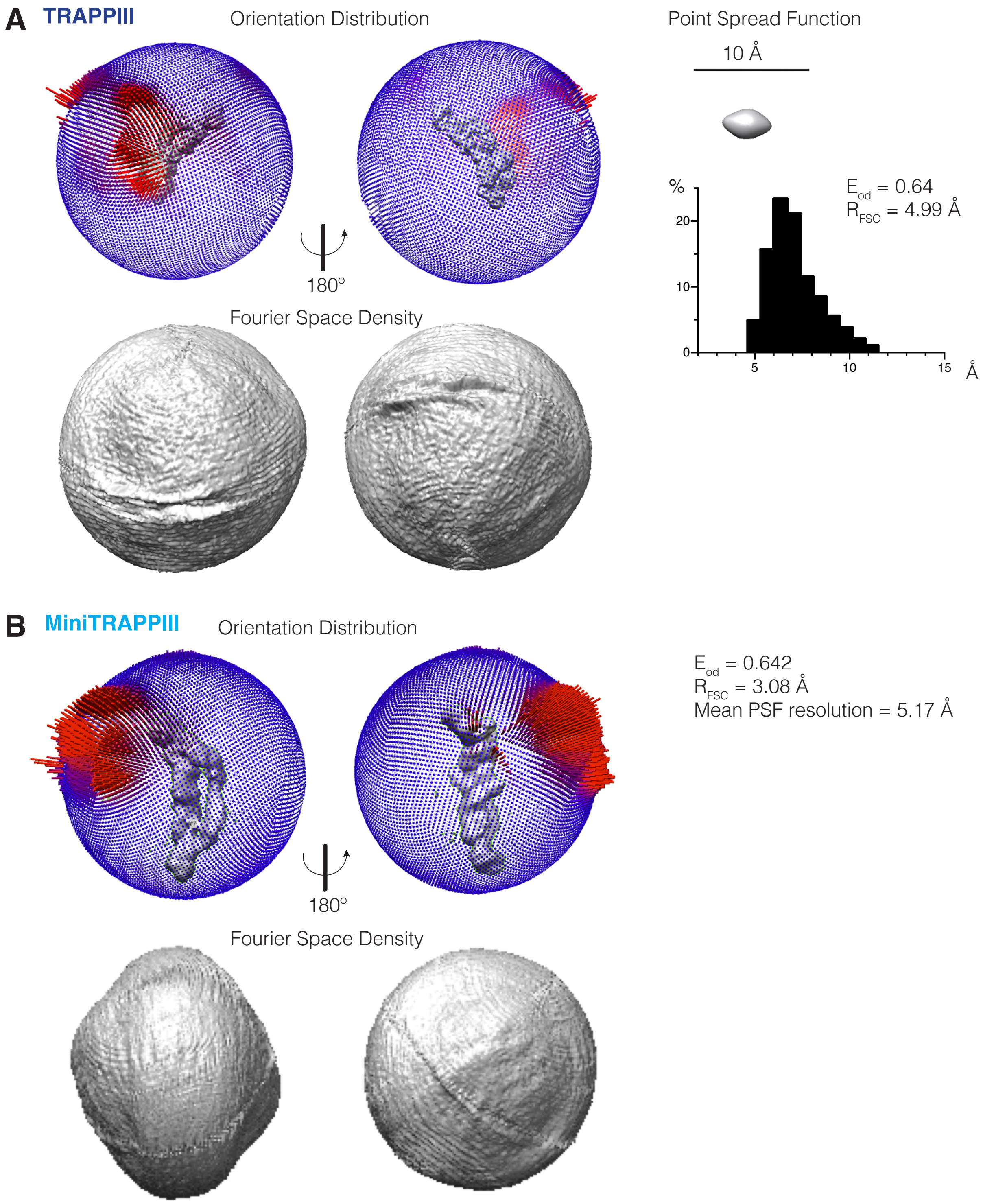

Figure S2. Angular distribution of non-tilted TRAPPIII and miniTRAPPIII.
(A) and (B). Particle orientation distributions for TRAPPIII and miniTRAPPIII quantified by calculating their efficiency, E_{od} (Naydenova & Russo, 2017).

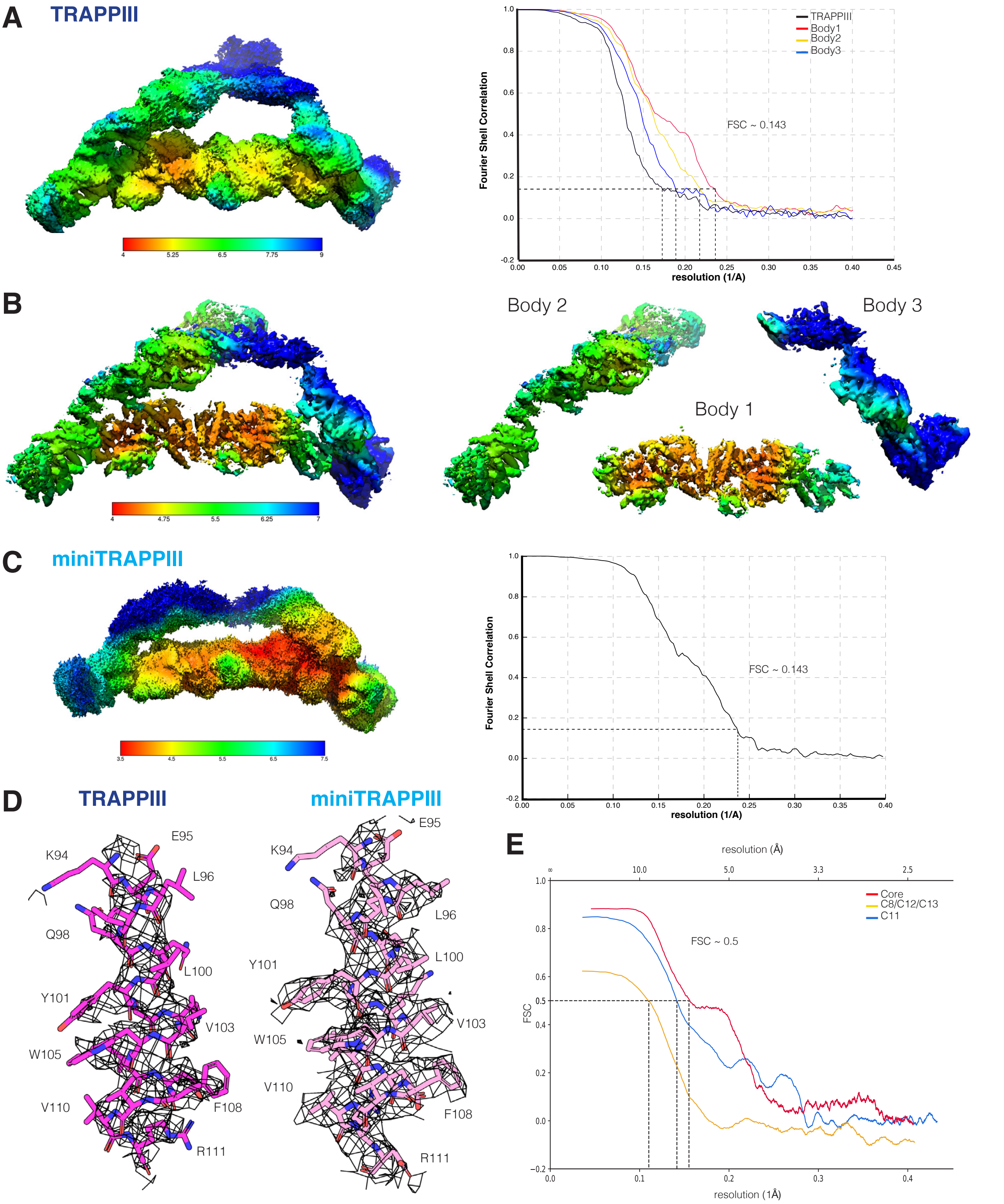

Appendix Figure S3. Cryo-EM structure of TRAPPIII and miniTRAPPIII.

(A) TRAPPIII particles from two non-tilted and two tilted data sets were merged and 3D refinement was performed with RELION 3.1 to yield consensus maps at a nominal resolution of 5.8 Å. This map was improved by multi-body refinement. Shown is the local resolution estimation of the resulting cryo-EM map, and FSC curves indicating the overall nominal resolutions using the FSC = 0.143 criterion (body1/core ~ 4.2 Å, body2/C8-C12-C13 ~ 4.5 Å, body3/C11 -5.4 Å).

(B) Local resolution estimation of the three different bodies used for multibody refinement.

(C) A data set of MiniTRAPPIII particles refined with RELION 3.1 to yield a consensus map at nominal resolution of 4 Å. The local estimation of the cryo-EM map and the FSC curve are shown.

(D) Cryo-EM densities and models for representative regions of TRAPPIII and miniTRAPPIII.

(E) FSC validation of the model versus cryo-EM map. The models refined against the first half-map (work), and the latter models versus the second half-map (free). The intersections with FSC=0.5 are shown.

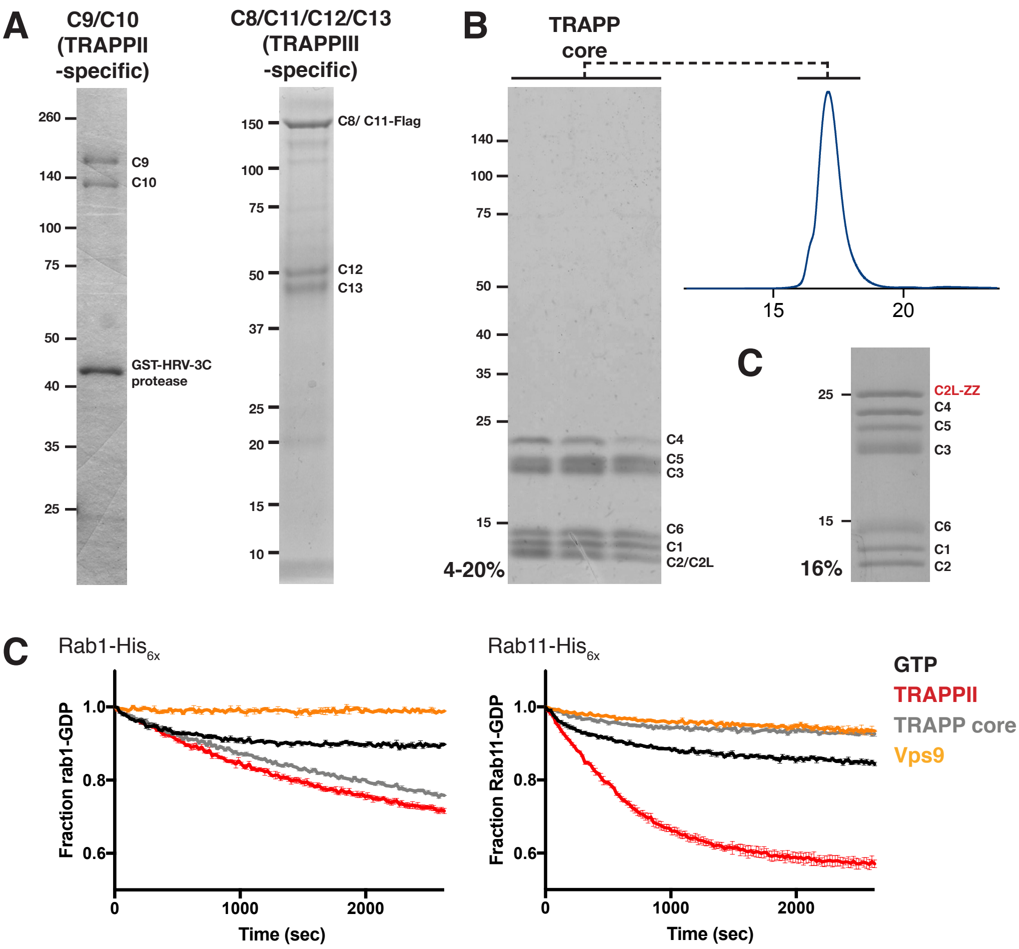

Appendix Figure S4. TRAPP subcomplexes.

(A) Coomassie blue-stained protein gels of purified TRAPP subcomplexes. FLAG tags on TRAPPC10 or TRAPPC11 allowed isolation of complexes comprised of the TRAPPII- or TRAPPIII-specific subunits, respectively, in the absence of core subunits. Complexes were eluted from anti-FLAG beads using either HRV-3C protease (TRAPPII) or SDS sample buffer without reducing agent (TRAPPIII).

(B) Coomassie blue-stained protein gel of purifief TRAPP core. TRAPPC3 was tagged with a Strep-Tag and the complex released from beads with GST-HRV-3C protease, with the latter removed using glutathione-Sepharose prior to size exclusion chromatography (SEC). UV trace from SEC is shown with all three fractions collected from the peak shown on a 4-20% gel.

(C) Coomassie blue–stained protein gels of TRAPP core purified using TRAPPC2L tagged with a ZZ domain, and eluted from IgG-Sepharose beads with low pH. Separated on a 16% gel to illustrate that all eight subunits are in the complex.

(D) GEF assays performed in triplicate. Rabs were loaded with mant-GDP, and the change in fluorescence was measured over time after addition of either the TRAPP core or complex TRAPPII. The Rab5 GEF Vps9 was used as a negative control.

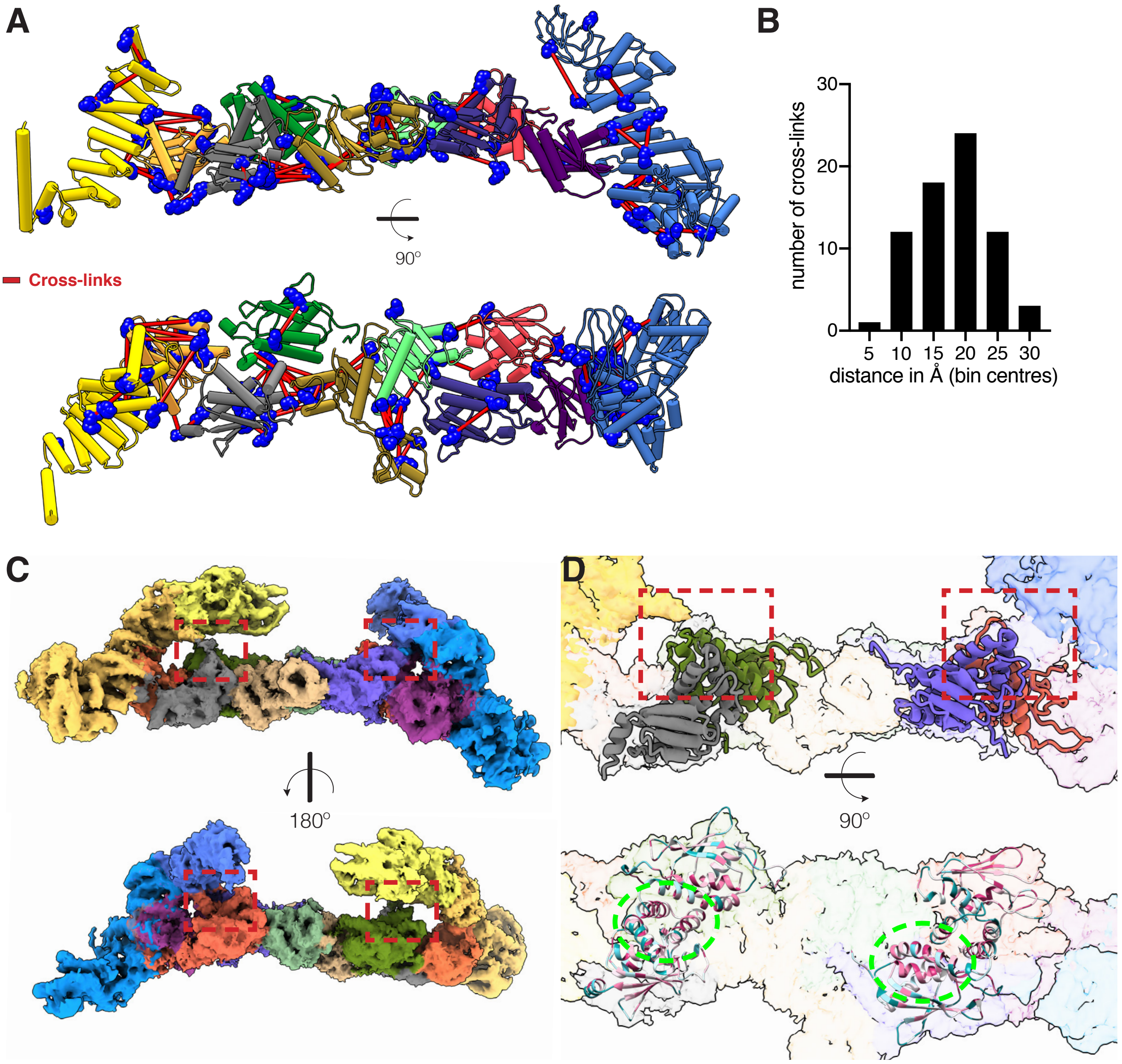

Appendix Figure S5. Cross-links and the TRAPPIII structural model.

(A) All cross-links falling within the expected Cα-Cα maximum distance for DSBU are mapped onto the structural model as red lines. Subunits differentiated by colour (C1: light green, C2: orange, C2L: magenta, C3a: purple, C3b: dark green, C4: light brown, C5: grey, C6: red, C8: yellow, C11: blue). Lysines in the structures indicated as blue dots.

(B) Distribution of Cα-Cα distances of DSBU cross-links (maximum distance ~30 Å).

(C) The TRAPPIII density map coloured according to different subunits (C1: light green, C2: orange, C2L: magenta, C3a: purple, C3b: dark green, C4: light brown, C5: grey, C6: red, C8: yellow, C11: blue). The distal parts of the TRAPPC8 and TRAPPC11 arms have been removed for clarity. The density linking the core with TRAPPC8 and TRAPPC11 is indicated by a red dashed square.

(D) Zoom of (C) showing the structural models of TRAPPC3a and TRAPPC6, and TRAPPC3b and TRAPPC5 fitted into the density near the bridges between the core and the arms. Subunits coloured as in (C). In bottom panel the subunits are coloured according to evolutionary conservation (red, most and blue, least). The part of the core that contacts the corresponding arm is formed by an arrangement of four α helices that is partially conserved (green dashed oval).

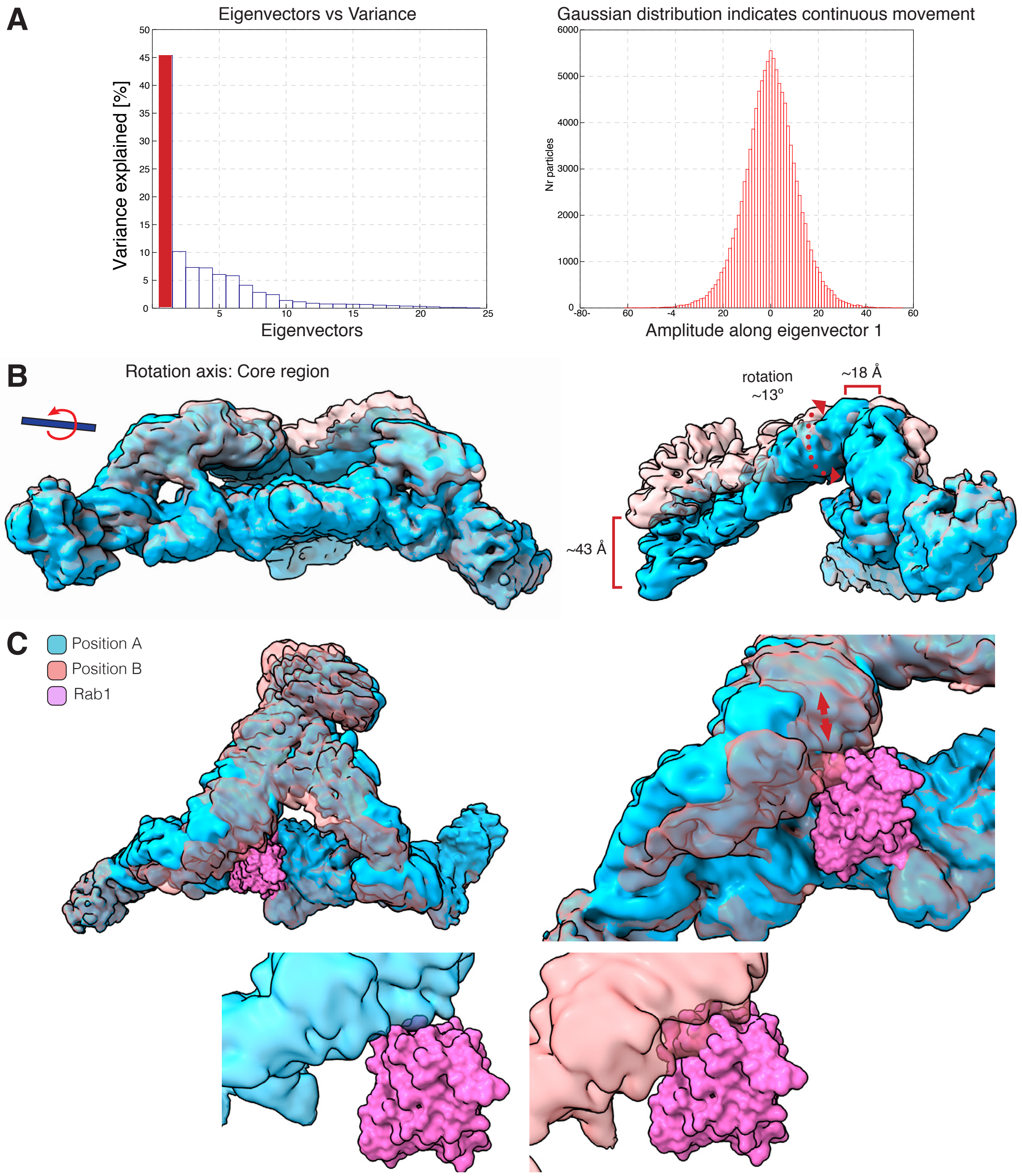

Appendix Figure S6. Movement of the TRAPPC8 and TRAPPC11 arms relative to the core.

(A) Results of the principal component analysis derived from the multibody refinement (Nakane et al, 2018). A single eigenvector accounts for almost half of the variance in the movement of the TRAPPIII complex relative to a body defined by the core and the N-terminal regions of TRAPPC8 and TRAPPC11. The histogram shows a unimodal distribution of the amplitude along this eigenvector, indicating a continuous range of positions.

(B, C) Motion represented by the main eigenvector from multi-body analysis in (A). The vector represents a rocking motion of the core region relative to the rest of the complex. This results in a rotation of the arms relative to the core of 13°, and a distance of around 18 Å between the two extreme positions of the part of TRAPPC8 that is predicted to touch Rab1 bound to the catalytic site.
