# Supplementary material for: Cryo‐EM structure of metazoan TRAPPIII, the multi‐subunit complex that activates the GTPase Rab1
Source: EMBO J. 2021 May 21;40(12):e107608. doi: 10.15252/embj.2020107608 (PMC8204870; doi:10.15252/embj.2020107608)
Supplement: Supplementary file 2 — Expanded View Figures PDF [file EMBJ-40-e107608-s006.pdf]

Expanded View Figures

Figure EV1. Characterisation of the TRAPP complexes.

A Coomassie blue-stained protein gels of recombinant *Drosophila* TRAPP complexes. The different TRAPP subunits are indicated.
B Normalised UV traces from SEC of the TRAPP complexes.
C iSCAT analysis of 50 nM samples of TRAPPIII, TRAPPII and miniTRAPPIII. Movies for each sample were recorded and their contrast translated into histograms. The histograms were fitted to a Gaussian distribution and compared with measurements from BSA standards to convert contrast into molecular mass (Cole *et al*, 2017) (TRAPPIII $\approx$ 548.5 kDa; TRAPPII $\approx$ 440 kDa; miniTRAPPIII $\approx$ 455 kDa).
D Determination of native molecular weight of TRAPPIII and miniTRAPPIII by SEC-MALS.
E GEF assays. Rabs were loaded with mant-GDP, and the change in fluorescence was measured over time after addition of either GTP alone (GTP) or GTP with the indicated GEF.

Source data are available online for this figure.

 © 2021 The Authors

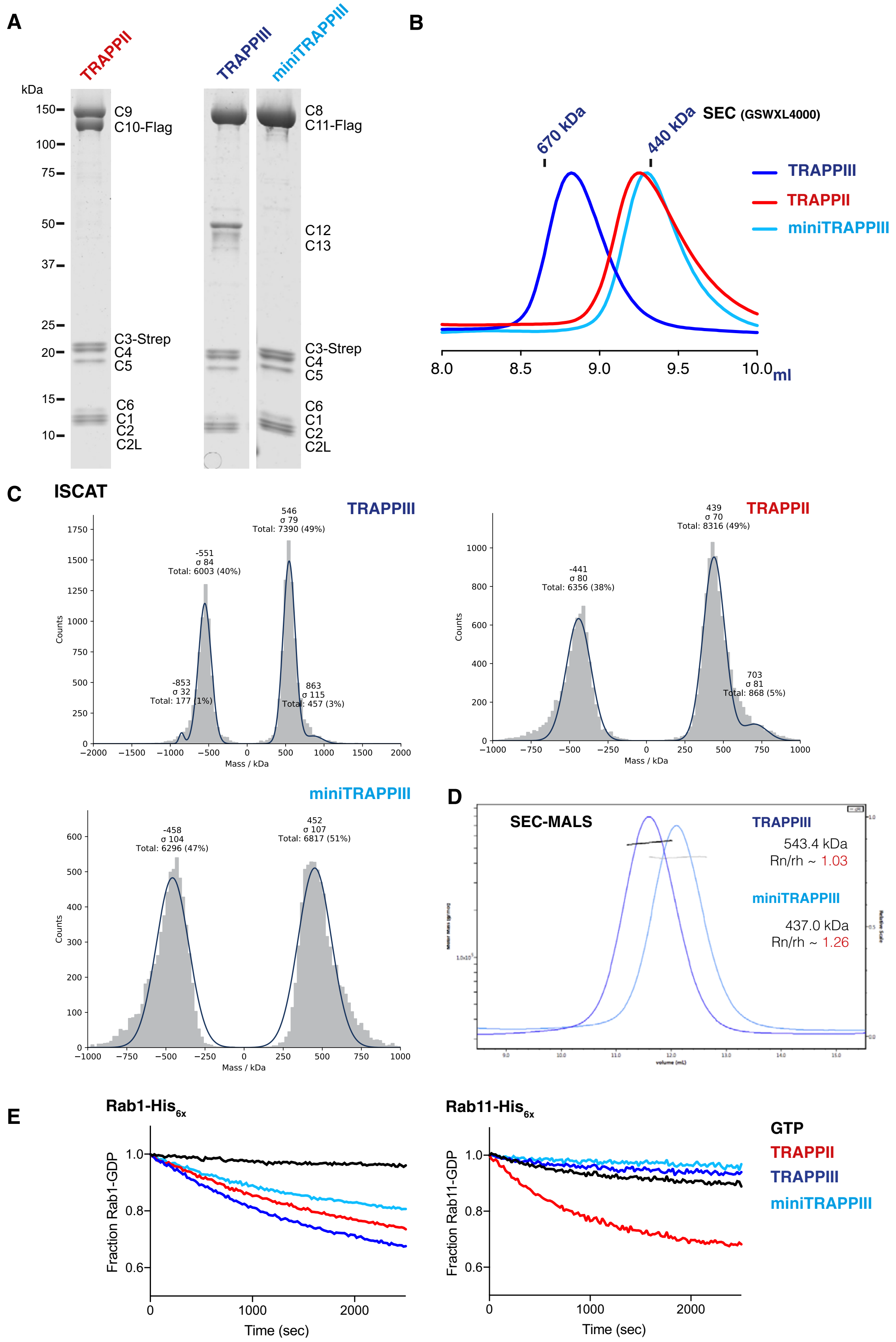
A
TRAPPII
TRAPPIII
miniTRAPPIII
kDa
150
100
75
50
37
25
20
15
10
C9
C10-Flag
C8
C11-Flag
C12
C13
C3-Strep
C4
C5
C6
C1
C2
C2L
B
670 kDa
440 kDa
SEC (GSWXL4000)
TRAPPIII
TRAPPII
miniTRAPPIII
8.0
8.5
9.0
9.5
10.0
ml
C
ISCAT
Counts
Mass / kDa
546
σ 79
Total: 7390 (49%)
-551
σ 84
Total: 6003 (40%)
-853
σ 32
Total: 177 (1%)
863
σ 115
Total: 457 (3%)
439
σ 70
Total: 8316 (49%)
-441
σ 80
Total: 6356 (38%)
703
σ 81
Total: 868 (5%)
-458
σ 104
Total: 6296 (47%)
452
σ 107
Total: 6817 (51%)
D
SEC-MALS
TRAPPIII
543.4 kDa
Rn/rh ~ 1.03
miniTRAPPIII
437.0 kDa
Rn/rh ~ 1.26
E
Rab1-His6x
Rab11-His6x
Fraction Rab1-GDP
Fraction Rab11-GDP
Time (sec)
GTP
TRAPPII
TRAPPIII
miniTRAPPIII

Figure EV1.

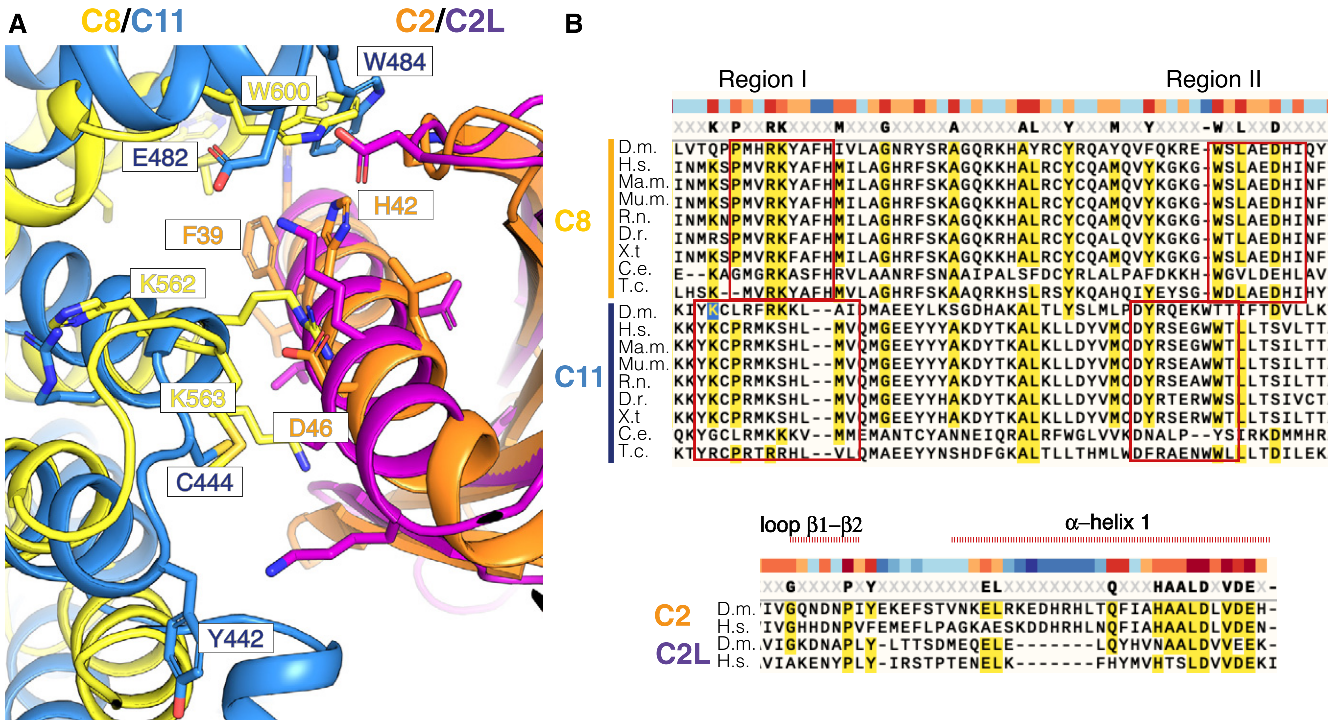

Figure EV2. Comparison of the TRAPPC2-TRAPPC8 and the TRAPPC2L-TRAPPC11 interfaces.

A The interface between TRAPPC2 and TRAPPC8 overlayed with that of TRAPPC2L and TRAPPC11. Residue numbers shown in the colour allocated to each subunit. TRAPPC2 and TRAPPC2L adopt a very similar fold (Fig EV3B), and a similar part of each abuts the helixes of TRAPPC8 and TRAPPC11.

B Alignments of the regions of TRAPPC8 and TRAPPC11 involved in the interactions with TRAPPC2 and TRAPPC2L respectively, and also of the regions of TRAPPC2 and TRAPPCL2 involved in the interactions. Bars at the top indicate the degree of conservation; red boxes show for TRAPPC8 the parts of the sequence displayed in Fig 3D, and for TRAPPC11 the parts shown in Fig 4D.

© 2021 The Authors

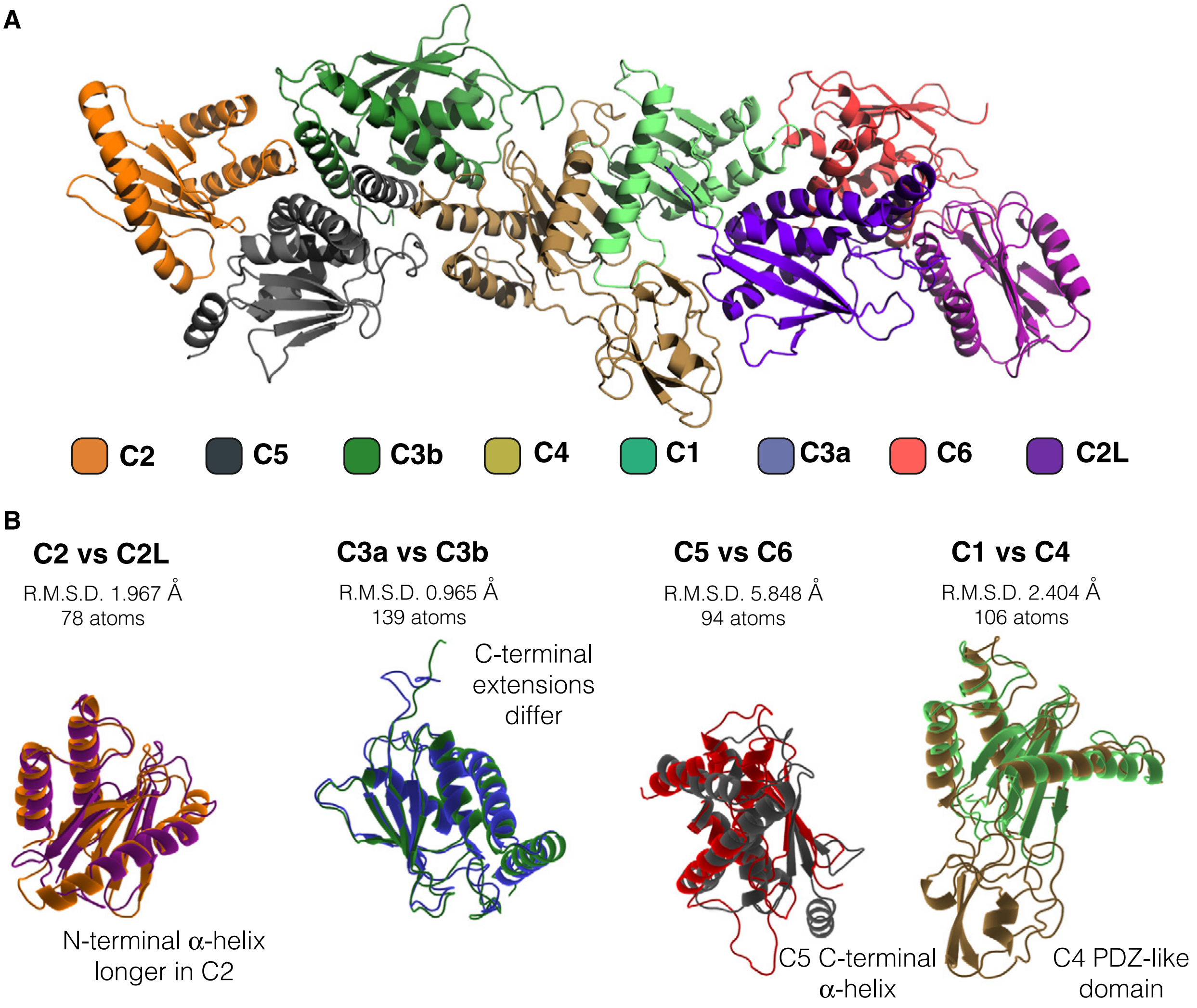

Figure EV3. Arrangement of TRAPP core subunits.

A Model of the core as fitted into the density map, with the eight subunits indicated by colour.

B Comparison of the related pairs of subunits indicating their close similarity, with labelling to describe the additional features present in only one of the pair.

Figure EV4. Architecture of the *Drosophila* TRAPPII complex.

A Top: representative 2D class averages of TRAPPII. The core region is indicated. Bottom: low-resolution cryo-EM map of TRAPPII.

B Circos-XL plot showing the distribution of all DSBU cross-links for the whole TRAPPII complex. Each protein is represented as a coloured segment (core subunits, red; TRAPPC9, yellow; TRAPPC10, purple), with residues numbers indicated. The relative position of the cross-link represents its location within the primary sequence. Inter-molecular cross-links are depicted as purples lines on the outside of the plot and intra-molecular cross-links as green lines inside of the plot.

C Network maps depicting the cross-links found for TRAPPC9 (light yellow) and TRAPPC10 (purple) in TRAPPII, and TRAPPC8 (dark yellow) and TRAPPC11 (blue) in TRAPPIII. The cross-links are coloured according to the MeroX Score. The cross-links to residue 47 in TRAPPC2L are highlighted by dotted lines.

Source data are available online for this figure.

© 2021 The Authors

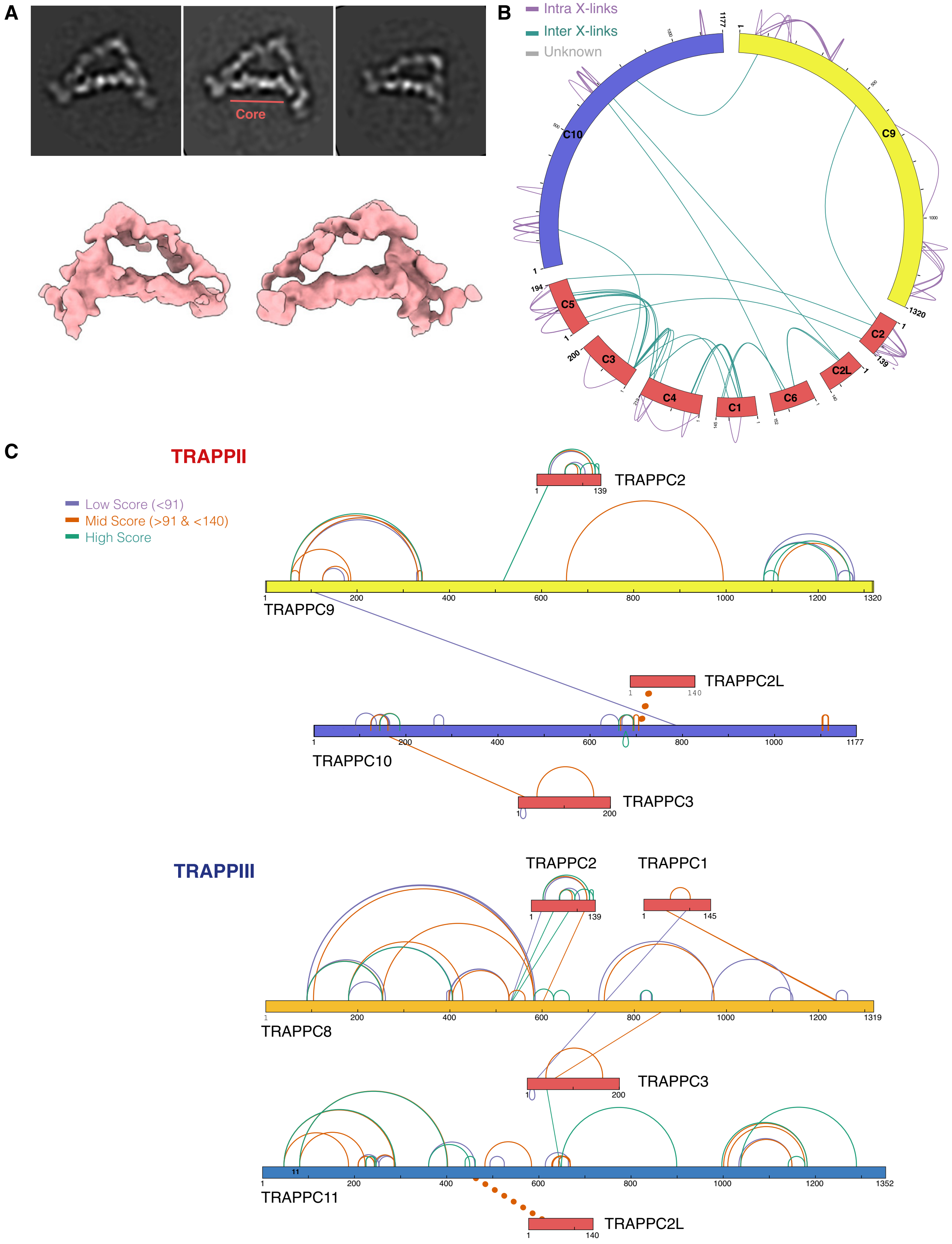

Figure EV4.

 © 2021 The Authors
