# Supplementary material for: Cryo‐EM structure of metazoan TRAPPIII, the multi‐subunit complex that activates the GTPase Rab1
Source: EMBO J. 2021 May 21;40(12):e107608. doi: 10.15252/embj.2020107608 (PMC8204870; doi:10.15252/embj.2020107608)
Supplement: Supplementary file 4 — Movie EV1 [file EMBJ-40-e107608-s003.zip › Legend_to_Movie_EV1.docx]

**A cryo-EM structure of metazoan TRAPPIII, the multisubunit complex that activates the GTPase Rab1**

Antonio Galindo*, Vicente J. Planelles-Herrero, Gianluca Degliesposti and Sean Munro

**Legend for Movie EV1.**

**Movie EV1.** Movement of the arms relative to the core as defined by the principal vector of flexibility identified by multibody refinement.
